# Defects in the COG complex and COG-related trafficking regulators affect neuronal Golgi function

**DOI:** 10.3389/fnins.2015.00405

**Published:** 2015-10-27

**Authors:** Leslie K. Climer, Maxim Dobretsov, Vladimir Lupashin

**Affiliations:** ^1^Department of Physiology and Biophysics, College of Medicine, University of Arkansas for Medical SciencesLittle Rock, AR, USA; ^2^Department of Anesthesiology, College of Medicine, University of Arkansas for Medical SciencesLittle Rock, AR, USA

**Keywords:** conserved oligomeric Golgi complex, COG, congenital disorders of glycosylation, neurodegeneration, glycosylation, vesicular trafficking, Rab, SNARE

## Abstract

The Conserved Oligomeric Golgi (COG) complex is an evolutionarily conserved hetero-octameric protein complex that has been proposed to organize vesicle tethering at the Golgi apparatus. Defects in seven of the eight COG subunits are linked to Congenital Disorders of Glycosylation (CDG)-type II, a family of rare diseases involving misregulation of protein glycosylation, alterations in Golgi structure, variations in retrograde trafficking through the Golgi and system-wide clinical pathologies. A troublesome aspect of these diseases are the neurological pathologies such as low IQ, microcephaly, and cerebellar atrophy. The essential function of the COG complex is dependent upon interactions with other components of trafficking machinery, such as Rab-GTPases and SNAREs. COG-interacting Rabs and SNAREs have been implicated in neurodegenerative diseases like Alzheimer's disease and Parkinson's disease. Defects in Golgi maintenance disrupts trafficking and processing of essential proteins, frequently associated with and contributing to compromised neuron function and human disease. Despite the recent advances in molecular neuroscience, the subcellular bases for most neurodegenerative diseases are poorly understood. This article gives an overview of the potential contributions of the COG complex and its Rab and SNARE partners in the pathogenesis of different neurodegenerative disorders.

## Introduction

The Conserved Oligomeric Golgi (COG) complex is an evolutionarily conserved hetero-octameric protein complex that is a proposed membrane tether during vesicular trafficking at the Golgi apparatus (Lupashin and Ungar, 2008; Reynders et al., 2011; Miller and Ungar, 2012; Willett et al., 2013b). COG is composed of two functionally distinct sub-complexes lobe A (COG1-4) and lobe B (COG5-8) (Fotso et al., 2005; Ungar et al., 2005). Secretory and transmembrane proteins make up 30–50% of all cellular proteins, and are trafficked through the endoplasmic reticulum (ER) to the Golgi for folding and modifications before delivery to their final destination. Secretory cargo molecules are thought to travel through the Golgi complex mostly inside flat cisternae that are constantly maturing in a *cis*-to-*trans* (anterograde) fashion via the so called cisternal maturation mechanism (Glick and Malhotra, 1998). However, resident Golgi proteins and Soluble NSF Attachment protein Receptors (SNAREs) are constantly recycled back in vesicular carriers to replenish the content of newly formed cis-cisternae. COG regulates the recycling of vesicles containing glycosylation enzymes and other resident Golgi proteins in a *trans*-to-*cis* (retrograde) direction. An intricate assortment of trafficking machineries including small Rab-GTPases, SNAREs, Sec1/Munc18 (SM) proteins, vesicular coat proteins, and tethering proteins are required for vesicular transport (Bonifacino and Glick, 2004). Intracellular pathways rely on these protein families at each step of vesicular transport. Though the functional interaction between the tethers and other trafficking regulators is not completely understood, a multi-subunit tethering complex (MTC), like the COG complex, may coordinate the interactions between all other components of the trafficking machinery at the site of vesicle docking on the target membrane for efficient fusion of the two membranes (Cottam and Ungar, 2012; Willett et al., 2013b).

SNAREs are an essential COG partner. SNAREs catalyze the fusion of the vesicle membrane with the target membrane by the assembly of a quaternary SNARE complex that functions as a zipper to coalesce the opposing lipid bilayers While SNAREs alone have an innate ability to fuse membranes, the SNARE regulatory proteins and tethering complexes are thought to be necessary for physiologically relevant fusion events (Rizo and Südhof, 2012). The COG complex interacts with at least two Golgi SNARE complexes: the *cis*-Golgi STX5/GOSR1(GS28)/YKT6/BET1L(GS15) complex; and the *trans*-Golgi STX16/STX6/VTI1a/VAMP4 complex (Shestakova et al., 2007; Laufman et al., 2009, 2013b). Additionally, SNARE complexes that contain Sec22b, GOSR2(GS27), or SNAP29 are evidenced to interact with COG (Kudlyk et al., 2013; Willett et al., 2013a). SNARE partner transitions connect all compartments of the endocytic and secretory pathways. Thus, it is likely that a defect in one trafficking step may have a cascading effect on all other cargo trafficking steps. In this review we will focus on those SNAREs where deficiency was shown to be associated with neuronal abnormalities, i.e., Ykt6, Sec22b, STX5, SNAP29, GS27, GS28, and Vti1a/b (Table 1).

**Table 1:** **Neurological phenotypes associated with COG and COG-interacting Rabs and SNAREs**.

**Protein**	**Disorder**	**Associated neurological manifestation**	**References**
**COG PROTEINS**			
COG1	CDG-IIg (COG1-CDG)	Cerebral atrophy, developmental delay, hypotonia	Foulquier et al., 2006
COG2	CDG-II (COG2-CDG)	Developmental delay, epilepsy	Kodera et al., 2015
COG4	CDG-IIj (COG4-CDG)	Developmental delay, epilepsy, hypotonia, lack of speech, nystagmus	Reynders et al., 2009; Ng et al., 2011
COG5	CDG-IIi (COG5-CDG)	Ataxia, cerebral atrophy, developmental delay, epilepsy, hypotonia	Paesold-Burda et al., 2009; Fung et al., 2012; Rymen et al., 2012
COG6	CDG-IIl (COG6-CDG) Shaheen syndrome (SHNS)	Ataxia, cerebral atrophy, developmental delay, epilepsy, hypotonia, optic nerve atrophy, sensoneural hearing loss Intellectual disability	Lübbehusen et al., 2010; Huybrechts et al., 2012; Shaheen et al., 2013; Rymen et al., 2015
COG7	CDG-IIe (COG7-CDG)	Cerebral atrophy, developmental delay, epilepsy, hypotonia	Wu et al., 2004; Morava et al., 2007; Ng et al., 2007; Zeevaert et al., 2009a
COG8	CDG-IIh (COG8-CDG)	Cerebral atrophy, developmental delay, hypotonia	Foulquier et al., 2007; Kranz et al., 2007
**SNARE PROTEINS**			
Ykt6	Parkinson's Disease	Trafficking defects and cytotoxicity *in vitro* in NRK and PC12 cell lines	Hasegawa et al., 2003, 2004
Sec22b	Parkinson's Disease	Trafficking defects and cytotoxicity *in vitro* in NRK and PC12 cell lines	Hasegawa et al., 2003, 2004
STX5	Parkinson's Disease Alzheimer's Disease	Trafficking defects and cytotoxicity *in vitro* in NRK and PC12 cell lines Regulates processing of APP in PC12, HeLa, COS-7, and NG108-15 cell lines and primary cultures of rat hippocampal neurons	Suga et al., 2005b; Thayanidhi et al., 2010; Rendón et al., 2013; Suga et al., 2015
SNAP29	CEDNIK-Neuro-cutaneous syndrome	Cerebral Dysgenesis, Neuropathy, Ichthyosis, and Keratoderma	Sprecher et al., 2005; Fuchs-Telem et al., 2011
GS27	Myoclonus epilepsy/early ataxia Parkinson's Disease	Action myoclonus, mild cerebral atrophy, and early ataxia Trafficking defects and cytotoxicity *in vitro* in NRK and PC12 cell lines	Thayanidhi et al., 2010; Corbett et al., 2011
GS28	Neurodegeneration	Retinal degeneration in *in vivo Drosophila* photoreceptors	Rosenbaum et al., 2014
Vti1a/b	Neurodegeneration	Perinatal lethality in double knockouts in an *in vivo* mouse model. Neuronal axon tracks missing, reduced in size or misrouted	Kunwar et al., 2011; Walter et al., 2014
**RAB PROTEINS**			
Rab1a	Parkinson's Disease	Neuroprotective in *C. elegans, D. melanogaster* and primary rat neuron cultures. Rescue from the neurotoxic effects of α-synuclein	Cooper et al., 2006; Gitler et al., 2008
Rab1b	Alzheimer's Disease	Dominant negative mutant of Rab1b blocks trafficking of APP and decreases the secretion of Aβ	Dugan et al., 1995
Rab2	Parkinson's Disease	Reduced expression of Rab2 can rescue Golgi fragmentation in PD models	Rendón et al., 2013
Rab4a	Neumann-Pick disease Alzheimer's Disease Down's syndrome	Reduced Rab4-dependent recycling *in vitro* in Neumann-Pick type A and type C fibroblasts. Postmortem samples: increased Rab4 in patients with AD and mild cognitive disorder Aβ partially co-localizes with Rab4 in a mouse model of Down Syndrome	Cataldo et al., 2000; Choudhury et al., 2004; Arriagada et al., 2010; Ginsberg et al., 2010
Rab6a	Alzheimer's Disease	Dominant negative mutant of Rab6 increases the secretion of soluble APP and decreased Aβ secretion	McConlogue et al., 1996

**Figure 1:**
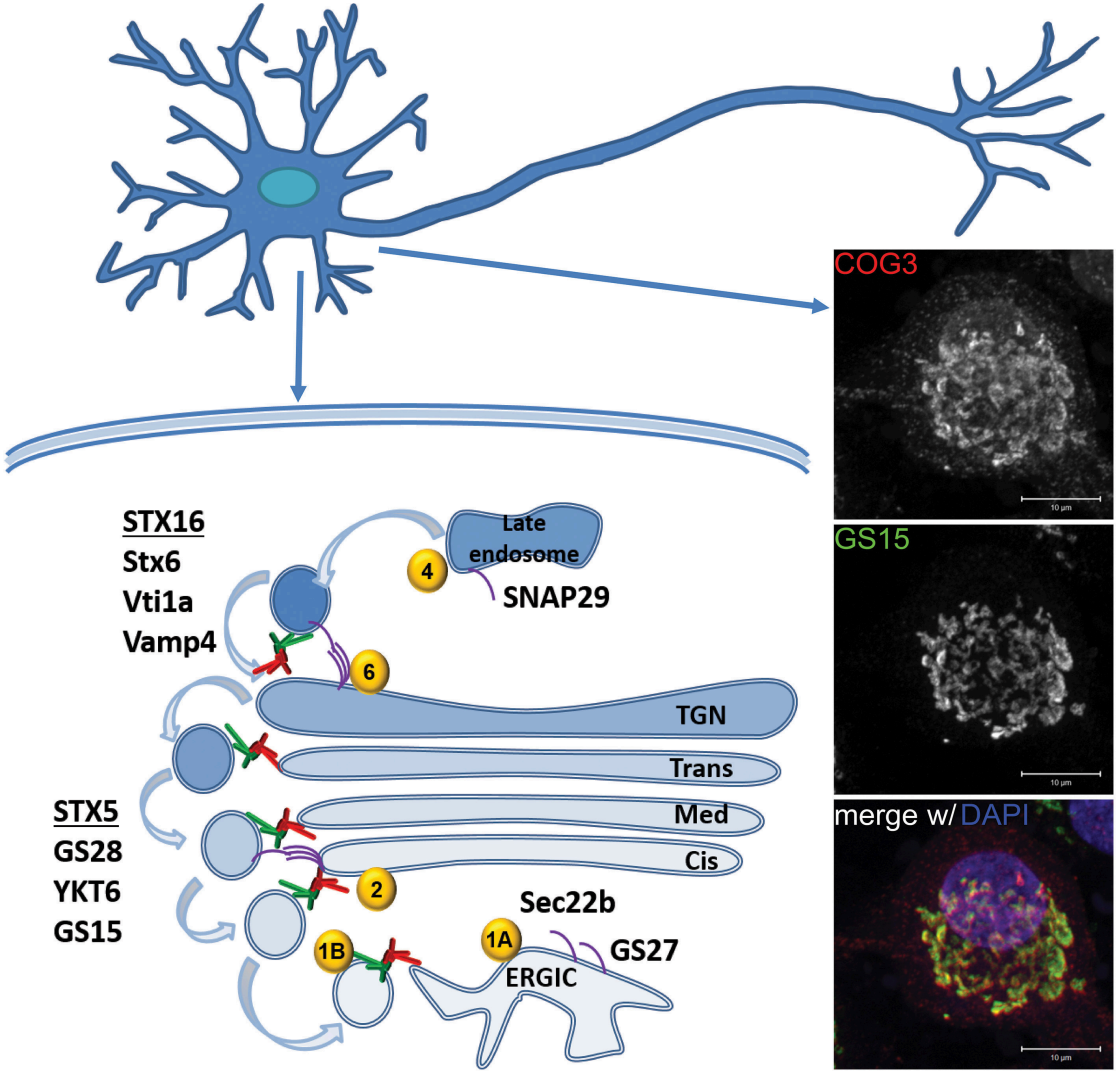
**Model of COG trafficking within neurons**. A cartoon depicting the proposed roles for the COG complex (depicted as red shapes for lobe A and green shapes for lobe B subcomplexes) and its interacting protein partners in Golgi trafficking: Rabs (yellow circles), and SNAREs (purple lines). Right panel: Immunofluorescence images of COG complex subunit COG3 (top-red) and Golgi SNARE GS15 (middle-green) in rat dorsal root ganglion. Perinuclear (DAPI) co-localization is indicated by yellow in the merged image (bottom). Scale Bar = 10 μm.

The essential function of the COG complex has been proposed to depend not only upon SNAREs interactions, but also interactions with Rabs (Miller et al., 2013; Willett et al., 2013a; Figure 1). Within this context, Rabs are believed to act as molecular switches that cycle between GDP-bound (inactive) and GTP-bound (active) states and regulate cargo trafficking by acting in an intracellular compartment-specific manner. Active (GTP-bound) Rabs regulate trafficking by binding effector molecules like tethering factors and motor proteins (D'Adamo et al., 2014). Binding to some effectors leads to the activation of other Rabs, in a sequence known as the Rab Cascade (Pfeffer, 2013). Nine distinct, Golgi-localized Rabs are known to interact with COG (Miller et al., 2013; Willett et al., 2013b, 2014). The compartment-specific nature of these different Rabs makes them a potential landmark for COG membrane localization and interaction. As with the SNAREs, this review will limit the discussion to COG partners Rab1a, Rab1b, Rab2, Rab4a, and Rab6a that are implicated in neuronal abnormalities (Table 1).

## Neurophysiological abnormalities and COG defects

Since 2004, defects in seven out of the eight COG subunits have been associated with human Congenital Disorders of Glycosylation (CDG)-type II, a growing family of diseases involving malfunctions in the processing of N- and O-linked glycans and resulting from mutations in proteins involved with glycosylation (Wu et al., 2004; Spaapen et al., 2005; Foulquier et al., 2006, 2007; Kranz et al., 2007; Ng et al., 2007, 2011; Paesold-Burda et al., 2009; Reynders et al., 2009; Richardson et al., 2009; Lübbehusen et al., 2010; Fung et al., 2012; Huybrechts et al., 2012; Rymen et al., 2012, 2015; Kodera et al., 2015; Table 1). Glycosylation is a highly dynamic process that occurs in the ER and Golgi which requires an estimated two percent of the human genome to encode the enzymes and trafficking components for the proper maturation of newly formed glycan chains (Freeze et al., 2014). COG deficiency can cause a redistribution of COG-dependent Golgi resident proteins, including glycosylation enzymes. Most COG-CDG patients have defects in sialylation and galactosylation, as indicated by fluorescent lectin staining of plasma membrane glycoconjugates from patient fibroblasts and MALDI-TOF mass spectrometry of serum glycoproteins (Foulquier et al., 2007; Kranz et al., 2007; Paesold-Burda et al., 2009; Reynders et al., 2009; Zeevaert et al., 2009a,b). Along with other multi-system pathologies, COG-CDG patients display mild to severe neurological defects including hypotonia, intellectual disability, developmental delays, epilepsy, and ataxia (Table 1). Specific symptoms and the severity of condition appears to relate to the COG subunit that is deficient with COG6 and COG7 patients demonstrating the most severe phenotypes (Rymen et al., 2015). Additionaly, COG defects have not yet been attributed to any other subtype of CDG.

Several CDGs result from mutated COG subunits that are either severely truncated or rapidly degraded. Loss of one COG subunit can destabilize the remaining subunits and reduce their expression and association with the Golgi. Early studies of the COG3 subunit invoked participation of the COG complex in the proper distribution of Golgi enzymes. COG3 depletion by siRNA in HeLa cells causes extensive Golgi fragmentation and destabilization of the COG complex (Zolov and Lupashin, 2005). COG3 and COG7 knockdown generates an accumulation of COG complex-dependent (CCD) vesicles carrying the SNAREs GS15 and GS28, and Golgi enzymes MAN2A1 and GALNT2 (Zolov and Lupashin, 2005; Shestakova et al., 2006). The accumulation of CCD vesicles suggests that in COG deficient cells a significant fraction of Golgi glycosylation enzymes are separated from the proteins they need to modify. COG8-CDG patient fibroblasts have decreased levels of the other lobe B subunits (COG5, COG6, and COG7) all of which have lost their association with the Golgi (Foulquier et al., 2007; Kranz et al., 2007). COG lobe B destabilization was also seen in COG7-CDG patient fibroblasts, resulting in the loss of COG6 association with the Golgi (Kudlyk et al., 2013). The loss of COG also challenges the function of interacting SNAREs. The endosome-to-*trans*-Golgi Network (TGN) SNARE protein STX16 was mislocalized in COG8-CDG patient fibroblasts (Willett et al., 2013a), and the STX5/GS28/Ykt6/GS15 and STX6/STX16/Vti1a/VAMP4 SNARE complexes were destabilized in both COG7- and COG8-CDG patient fibroblasts (Laufman et al., 2013a). In a non-CDG patient presenting intellectual disability, Shaheen et al. identified a mutation in COG6 which resulted in reduced COG6 and STX6 protein expression (Shaheen et al., 2013). Anterograde trafficking does not appear to be affected in cells with COG mutations, but retrograde trafficking is affected as indicated by a partial resistance to treatment with the transport inhibitor brefeldin A (Steet and Kornfeld, 2006; Foulquier et al., 2007; Kranz et al., 2007; Ng et al., 2007; Paesold-Burda et al., 2009; Reynders et al., 2009) and by endosome-to-TGN trafficking defects elucidated by application of Shiga toxin and SubAB toxin (Zolov and Lupashin, 2005; Smith et al., 2009). Therefore, retrograde intra-Golgi and endosome-to-TGN sorting are particularly impaired by COG deficiency.

## Neuropathology and defects in COG-associated proteins

Extensive *in vitro* analyses in control and disease models demonstrate that genetic deficiency in SNARE and Rab COG partners may also result in disintegration of the Golgi apparatus, thereby potentially influencing neurological impairment (Table 1). Interestingly, the therapeutic implications of studying neurodegeneration associated with defective COG function is broader than the COG-CDG patient population (D'Adamo et al., 2014; Rymen et al., 2015). Golgi fragmentation is a common feature of neurodegenerative diseases (Gonatas et al., 2006). Current theories argue that the Golgi fragmentation seen in Alzheimer's Disease (AD) and Parkinson's Disease (PD) is either a result of misfolded or aggregated proteins, or that fragmented Golgi causes etiologically important proteins to aggregate and misfold leading to further progression of these diseases (Gonatas et al., 2006; Nakagomi et al., 2008; Bellouze et al., 2014; Joshi and Wang, 2015). However, Golgi fragmentation has also been linked to SNARE and Rab proteins making it difficult to pinpoint a single disease progression. Thus, the COG-Rab-SNARE dynamic is important for understanding neurodegenerative phenotypes.

## SNAREs

Genetic deficiency in SNAREs was shown to be associated with the progression of neurodegenerative diseases like AD and PD. PD is marked by the presence of Lewy bodies which are principally composed of aggregated α-synuclein. Under physiological conditions, α-synuclein may regulate vesicle trafficking and promote synaptic transmission by binding directly to SNAREs and stimulating SNARE complex formation (Burré et al., 2010; Thayanidhi et al., 2010). Overexpression of wild type or the PD-associated mutant of α-synuclein (A53T) leads to cytotoxicity and inhibition of ER-to-Golgi trafficking in *in vitro* models which can be partially suppressed by co-overexpression of SNAREs Ykt6 or Sec22. Ykt6—a protein enriched in neurons—was more protective than Sec22 and suggests a specialized role in mammals (Hasegawa et al., 2003, 2004). *In vitro* binding experiments also point toward the direct interaction of α-synuclein with STX5 and GS27 along with mutant α-synuclein which destabilizes the STX5-GS27-rbet1-sec22b SNARE complex (Thayanidhi et al., 2010). This line of evidence further implicates trafficking defects in bringing about neurodegenerative cytotoxicity.

Although the exact molecular mechanisms connecting Golgi fragmentation and disease mutations is still much under investigation, *in vitro* models can be used to recapitulate Golgi fragmentation seen in neurodegenerative disorders (Suga et al., 2005a). Fragmentation has been recreated *in vitro* and can be rescued by regulating levels of SNAREs. A STX5 knockdown is known to induce Golgi fragmentation in HeLa cells and cultured neurons (Suga et al., 2005a; Amessou et al., 2007). On the other hand, in PC12 cells treated with 6-hydroxydopamine or methamphetamine (an established *in vitro* PD model), a decrease in the level of STX5 rescues Golgi fragmentation (Rendón et al., 2013). This same study also demonstrated that Golgi fragmentation could intensify disease progression by inducing α-synuclein aggregation and the formation of Lewy bodies.

STX5 defects have also been shown to affect processing of AD-related proteins. In AD, amyloid precursor protein (APP) undergoes a series of proteolytic events by β- and γ-secretases to create the amyloidogenic variants of β-amyloid (Aβ) that are longer and more likely to form aggregates (Peric and Annaert, 2015). Presenilins form a complex with γ-secretase, and mutations in presenilin 1 (PS1) are the most frequently associated mutations found in AD which result in increased production of Aβ, or altered ratios of amyloid peptide species (Hardy, 2006; Saito et al., 2011; De Strooper et al., 2012). Overexpressed STX5 was shown to co-localize with and directly bind to PS1. Further, STX5 overexpression increased the accumulation of APP in the ER and cis-Golgi and inhibited Aβ secretion in a neuroblastoma cell line (NG108-15)(Suga et al., 2005b). In NG108-15 cells expressing the familial AD mutation PS1ΔE9, STX5 was shown to have a decreased association with presenilin. A study of ER stress in an AD model demonstrated that ER stressors can increase synthesis of STX5 and its accumulation in the ER-Golgi intermediate compartment (ERGIC) and transport vesicles. Thus, upregulation of trafficking machinery induced by ER stress could be a cellular mechanism for correcting the accumulation of the amyloidogenic cleavage products of APP (Suga et al., 2015).

As stated above, the *cis*-Golgi SNARE GS27 (GOSR2) binds to α-synuclein and is part of a SNARE complex that is destabilized by mutant α-synuclein. GS27 has also been shown to be associated with a neurological disorder in humans. Six patient were identified baring a mutation that results in improper subcellular localization and loss of function of GS27 leading to symptoms common in COG-CDG patients such as cerebral atrophy, epilepsy, and early ataxia (Corbett et al., 2011; Table 1).

GS28 (GOSR1) is a Golgi SNARE involved in both ER-to-Golgi and intra-Golgi transport (Nagahama et al., 1996; Subramaniam et al., 1996), and accordingly has been shown to be associated with three SNARE complexes (Zhang and Hong, 2001; Parlati et al., 2002; Xu et al., 2002; Siddiqi et al., 2010). GS28 mutants have been used to study retinal degeneration in *Drosophila* photoreceptors (Rosenbaum et al., 2014). Lack of expression of GS28 in mutant flies alters trafficking and glycosylation of rhodopsin (Rh1). The photoreceptors in these mutants also exhibit enlarged ER and Golgi membranes and retinal degeneration over time.

Vti1a is a TGN-localized SNARE that functions in vesicle generation and Ca2+ channel trafficking (von Mollard et al., 1997; Lupashin et al., 1997; Walter et al., 2014). A double knockout of Vti1a and Vti1b genes results in progressive neurodegeneration and perinatal lethality in a mouse model (Kunwar et al., 2011). Single knockout of Vti1a or Vti1b does not result in a lethal phenotype indicating overlapping functions of these proteins. The death-after-birth clearly demonstrates that these SNAREs are not required during organismal development, indicating a specialized requirement for Vti1a and Vti1b in neurons leading to neurodegeneration in the double-knockout animals (Walter et al., 2014). Vti1a SNARE partners, STX6 and STX16, are also required for neurite outgrowth (Chua and Tang, 2008; Kabayama et al., 2008).

In humans, a disease known as CEDNIK (Cerebral Dysgenesis, Neuropathy, Ichthyosis, and Keratoderma) syndrome has been linked with loss of function mutations in SNAP29 (Sprecher et al., 2005; Fuchs-Telem et al., 2011). SNAP29 is a member of the SNAP25 family that localizes to the Golgi, endosomal, and lysosomal compartments (Steegmaier et al., 1998). CEDNIK patients exhibit severe neuropathy likely due to the loss of SNAP29 functional involvement in neurotransmission (Pan et al., 2005) and trafficking within neuroglia during active myelination (Schardt et al., 2009).

## Rabs

As for SNAREs, regulating levels of Rabs has been shown to rescue Golgi fragmentation in multiple *in vitro* models of AD and PD. For example, overexpression of Rab1 can rescue Golgi fragmentation while reduced expression of Rab2 has the same affect in PD models which demonstrates the delicate balance in the regulatory functions of Golgi-associated Rabs (Rendón et al., 2013). Overexpression of Rab1 was shown to be neuroprotective in *Caenorhabditis elegans, Drosophila melanogaster* and primary rat neuron cultures (Cooper et al., 2006; Gitler et al., 2008). Rab1 is a key protein in maintaining Golgi architecture and function (Haas et al., 2007). It can also promote the restoration of ER-to-Golgi trafficking and thus afford rescue from the neurotoxic effects of α-synuclein (Cooper et al., 2006; Gitler et al., 2008).

Multiple Rabs are associated with processing of APP. For example, ERGIC and *cis*-Golgi Rab1b-dependent trafficking could modulate the processing of APP as demonstrated in an *in vitro* system in which a dominant-negative mutant of Rab1b blocked trafficking of APP and decreased the secretion of Aβ (Dugan et al., 1995). *trans*-Golgi Rab6A is also implicated in APP trafficking. The dominant negative mutant of Rab6 increased the secretion of soluble APP and decreased Aβ secretion (McConlogue et al., 1996). Rab6 membrane association is dependent upon PS1 based on studies using PS1 knockout fibroblasts, thus implicating PS1 in vesicular trafficking (Scheper et al., 2004).

Golgi-associated and endosomal Rab4 is important for a specific pool of constitutively recycling endosomes which are apparently critical for dendritic spine size. Rab4-dependent recycling is greatly reduced in fibroblasts of patients with type A/B Niemann-Pick disease, a sphingolipid storage disorder which commonly manifests with neurological symptoms such as developmental delay and dementia (Choudhury et al., 2004). Postmortem samples revealed that Rab4 is upregulated in patients with AD and mild cognitive disorder (Cataldo et al., 2000; Ginsberg et al., 2010), and Aβ is known to partially co-localize within Rab4 positive compartments in a mouse model of Down Syndrome (Arriagada et al., 2010) indicating that defects in endosomal sorting may underpin these disorders (Peric and Annaert, 2015).

## Conclusions and perspectives

The COG complex and its Rab and SNARE partners are evolutionarily conserved and ubiquitously expressed across multiple tissues and organ systems in humans. Yet, neurological symptoms are the most debilitating and troublesome clinical manifestations of COG-associated disorders. Why is this the case? We propose that brain-specific manifestations of COG defects result from either COG-dependent (a) glycosylation defects and/or, (b) trafficking defects and/or, (c) a yet unknown neuron/neuroglia-specific function of the COG complex and its partners.

Neuronal function critically depends on coordinated delivery of properly modified ion channels, transporters and components of the synaptic apparatus at the appropriate rates and over long distances, to specific subcellular compartments. Remarkably, the localization of the synaptobrevin homolog Snc1 is altered in COG deficient yeast cells (Whyte and Munro, 2002). In addition, underglycosylated low density lipoprotein receptor is severely destabilized in CHO cells deficient for COG1 or COG2 proteins (Kingsley et al., 1986). Since glycosylation of channels, transporters and transport regulators is essential for their correct delivery, stability and/or activity (Gong et al., 2002; Watanabe et al., 2004; Scott and Panin, 2014), it is reasonable to predict that a majority of underglycosylated ion channels and transporters may similarly be destabilized, thus altering the functionality of COG-CDG patient neurons.

Smooth transport of cargo by the trafficking machinery is very important during development and synaptic transmission. Defective glycosylation of proteins and lipids disrupts development pathways and alters brain function (Freeze et al., 2012). COG deficient cells also display altered glycosphingolipid biosynthesis. Complex gangliosides are sialic acid containing glycosphingolipids synthesized sequentially, beginning with GM3 and then extended by glycosyltransferases to the more elaborate GM1 gangliosides. Biochemical studies revealed decreased levels of sphingomyelin and GM3 gangliosides in COG2 deficient CHO cells (Spessott et al., 2010a,b). Gangliosides are ubiquitously expressed, but in the brain the expression of gangliosides and their glycosyltransferases change dramatically during development, from an abundance in of the precursor GM3 to a greater abundance of GM1 (Yu et al., 1988; Kracun et al., 1991, 1992; Ngamukote et al., 2007). Therefore, altered glycosphingolipid biosynthesis could be another reason for the specific neurological manifestation in COG-related congenital disorders.

An additional factor that may add to the apparently higher neuronal vulnerability to COG deficiency is that unlike many other cells, neurons are non-dividing cells and hence cannot easily dilute toxic proteins, peptides, and organelles. Another possibility is that COG is playing a specific and yet uncovered role in neuronal cells. Although the COG complex has been largely detected at the established perinuclear Golgi location in neurons (Figure 1) this does not exclude some sort of moonlighting function for COG subunits at other neuron-specific locations where it could be involved in tethering of specialized vesicles.

Considering the systemic and complex character of COG-related diseases, multiple questions regarding the associated neurological symptoms remain to be addressed. Thus, more *in vivo* studies are needed to dissect the role of COG and its Rab and SNARE partners in these symptoms. Input from COG deficiencies within neuroglia and subtypes of neuroglia presents yet another poorly studied field and avenue requiring further studies. Further research is also needed to establish in which diseases COG-deficiency-associated neurological syndromes are secondary manifestations of some other primary disease state.

### Conflict of interest statement

The authors declare that the research was conducted in the absence of any commercial or financial relationships that could be construed as a potential conflict of interest.
